# Resting behavior of *Aedes aegypti* in southeastern Senegal

**DOI:** 10.1186/s13071-020-04223-x

**Published:** 2020-07-18

**Authors:** Diawo Diallo, Mawlouth Diallo

**Affiliations:** Pôle de Zoologie Médicale, Institut Pasteur de Dakar, 36 Avenue Pasteur, BP 220 Dakar, Senegal

**Keywords:** *Aedes aegypti*, Resting places, Rooms, Tires, Bricks, Scrap metal, Southeastern Senegal

## Abstract

**Background:**

Only the sylvatic and zoophilic population of *Aedes aegypti* was formerly identified in southeastern Senegal. A newly established anthropophilic population was detected in the urban area of the Kedougou city. Because of its new behavior, this species could play a primary role in the transmission of dengue and other arboviruses in this area. Because these arboviruses have no vaccine and specific treatments, vector control remains the only effective way to control their outbreaks. Effective vector control strategies require to understand some aspects of the bioecology of the vector, specially resting behavior. The aims of this study were to investigate the sites and resting behavior of *Ae. aegypti* in southeastern Senegal.

**Methods:**

Mosquitoes were collected in several potential resting places (rooms, tires, bricks and scrap metal) by two technicians using a CDC back-pack aspirator in the Kedougou bus station and other sites within the city and the nearby rural area. Collected mosquitoes were identified and classified.

**Results:**

A total of 1291 mosquitoes belonging to 6 genera and 20 species were collected. *Aedes aegypti* was the dominant species in all the resting places investigated. This species was found resting equally in rooms, bricks, tires and scrap metal. The average number of *Ae. aegypti* collected in resting places was higher in the bus station (center of the city) compared to the other areas. The rates of unfed and fed females varied significantly in the different resting places while the proportions of gravid females which varied between 7.8% in tires and 1.8% in rooms were comparable.

**Conclusions:**

This study showed that *Ae. aegypti* could be found resting indoors and in several sites, including in used tires outdoors. These data will be helpful in setting better arboviruses surveillance and vector control strategies.
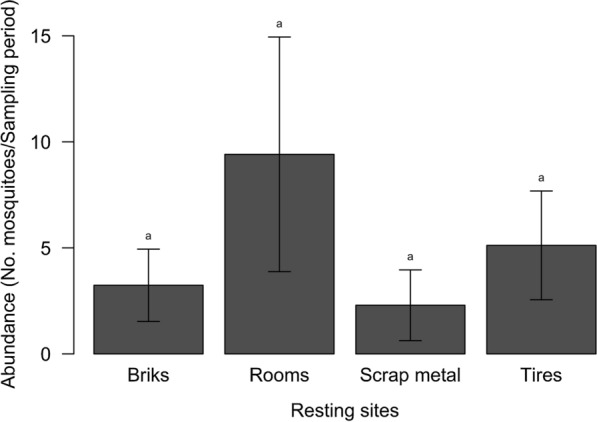

## Background

Dengue, chikungunya, Zika and yellow fever have experienced a global expansion and a considerable increase in their incidence worldwide during the last decades [1–4]. The arboviruses responsible for these emerging and/or re-emerging diseases have the particularity of being transmitted to humans mainly, or exclusively, by the mosquito *Aedes aegypti* in their urban cycle [5, 6].

Because these arboviruses have currently no specific treatment and no effective vaccine (except yellow fever), vector control remains the only control method available against them. Among vector control methods currently available, targeting the adult population by aerial spraying of insecticides is considered the most effective in reducing the female population and preventing and/or curbing arbovirus transmission [7]. However, the implementation of aerial spraying of insecticide for the control of adult mosquitoes requires, among others, knowledge of the resting behavior and sites of the population of *Ae. aegypti*. This species is generally considered, in many areas of the world, as being very anthropophilic, feeding and resting mainly inside human dwellings [8–11], although other authors have suggested that they could also probably be found resting outdoors near their breeding sites [12].

In Senegal, little is known on the bioecology of this species in general, and its sites and resting behavior in particular. The *Ae. aegypti* from Senegal, belonging to the *formosus* type, was considered to be composed of two subpopulations. A domestic and anthropophilic sub-population, feeding and resting mainly inside human dwellings, and a wild sub-population, zoophilic and resting in the forest environment far from human habitation [13–15]. The wild sub-population, which seemed to exist only in the forests of southeastern Senegal [16], was recently found in abundance in the larval breeding sites in the domestic environment, even if the species still feeds very little on humans, suggesting a process of progressive domestication [15]. As part of the surveillance of arbovirus vectors in southeastern Senegal, we found a very aggressive population for humans at the Kedougou bus station in the center of the city, suggesting the introduction of a new anthropophilic population or a progression of the domestication process of the wild population of *Ae. aegypti*, with a change in hosts preference from mammals to humans. It seemed important to us to study some aspects of bioecology of this population that could allow us to control or eradicate it in the area.

The objective of this study is to investigate the resting places and behavior of this neourban and anthropophilic population of *Ae. aegypti* from southeastern Senegal.

## Methods

### Study sites

**Fig. 1:**
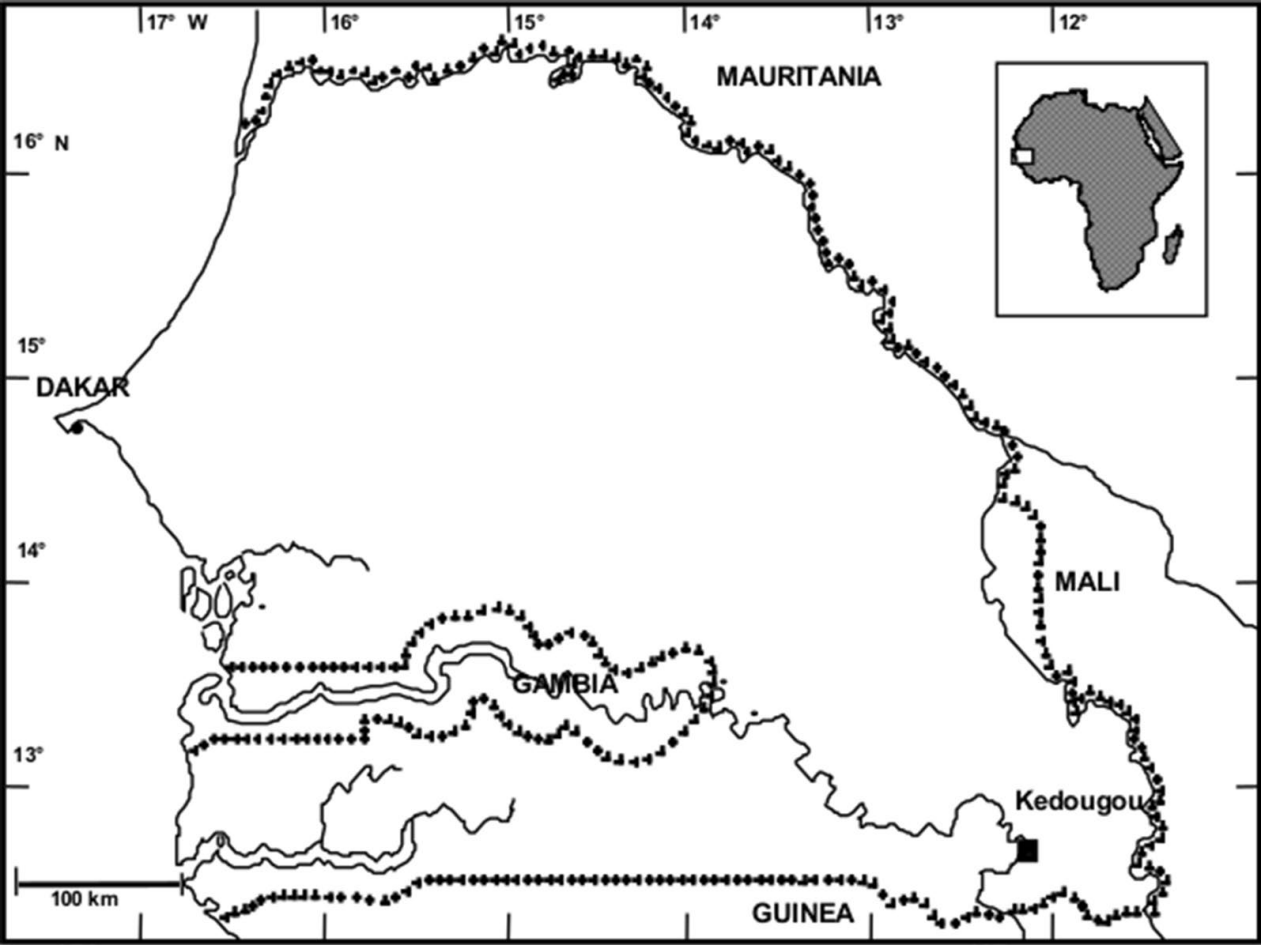
Study area

The study was carried out in Kedougou city (12°33′N, 12°11′W) and its surroundings, located in southeastern Senegal (Fig. 1), during the 2018 rainy season. This region belongs to the Sudano-Guinean climate, with a single rainy season which generally lasts 6 months (between May and October; with an average rainfall of 1200 mm) and average temperatures vary between 33 °C and 40 °C.

The Kedougou bus station was chosen as the main study site due to the detection of an *Ae. aegypti* population highly aggressive to humans in this site unlike the rest of the area. This bus station is characterized by the presence of numerous workshops with large quantities of used tires and abandoned vehicles outdoors. The site also has numerous restaurants, dwellings, and many bricks used for the construction of these buildings. Two other areas of the city (within the city), a farm and a village located less than 10 km from the city (rural area) were also sampled for comparison. The other areas within the city are characterized by dispersed dwellings and lot of houses in construction surrounded by many bricks (used for their construction), trees, and tall grasses. Fewer used tires and scrap metal are found in these areas compared to the bus station. The village and farm (rural area) are located in the savannah environment at the vicinity of several forest galleries. They presented fewer houses, used tires and bricks.

### Mosquito collection

The collection was carried out by two technicians using a CDC Back-Pack aspirator [17]. Mosquitoes were collected in each of the potential resting place identified in the area (rooms, tires, bricks and scrap metal) for a period of 30 min per place type with a relay between technicians each 15 min.

Collections were made, for 3 to 6 consecutive days per month at the bus station and 1–4 days per month for the other sites within the city (2 sites) and the rural area (2 sites), between August and November 2018. Overall, mosquitoes were collected during 34 sampling days, including 16 days at the bus station (6 days in August, 4 in September, 3 in October and 3 in November), 10 days at other sites within the city (1 day in August, 2 in September, 2 in October and 5 in November), and 8 days in the rural area (2 days in August, 2 in September, 2 in October and 2 in November) around the city.

All collected mosquitoes were cold-killed, identified following available keys [18–21] and classified by species, sex, physiological state and type of resting site and sampling origin.

### Data analysis

The average numbers of resting *Ae. aegypti* collected per unit of time (30 min) according to the resting place type, and the location of study were evaluated and compared using the Kruskal-Wallis test followed by the Dunn’s multiple comparison test for significant testing. Rates of unfed, fed and gravid females collected resting in the different resting places were compared using the Chi-square test. Statistical significance was set at *P* < 0.05. All data was analyzed using R software [22].

## Results

### Mosquito species composition and relative abundance in different resting places

A total of 1291 mosquitoes belonging to 20 species within 6 genera, including 5 species of *Aedes*, 5 species of *Anopheles*, 7 species of *Culex*, 1 species of *Mansonia*, 1 species of *Mimomyia* and 1 species of *Uranotaenia* were collected (Table 1). The dominant species were *Ae. aegypti* (52.8% of the mosquitoes collected) and *Cx. quinquefasciatus* (34.5%), followed, among species with more than 1% of the fauna, by *Cx. perfuscus* (3.1%), *An. gambiae* (2.1%), *Cx. nebulosus*, *Cx. decens* (1.9% each) and *Ae. vittatus* (1.2%). Mosquitoes were collected resting, in descending order, in rooms (*n* = 542; 42.0% of the mosquitoes collected), tires (*n* = 342; 26.5%), bricks (*n* = 263; 20.4%), and scrap metal (*n* = 144; 11.1%). *Aedes aegypti* was the most abundant species in all the resting places investigated, and represented 59%, 41.8%, 50.9% and 54.2% of the mosquitoes resting in rooms (320/542), bricks (110/263), tires (174/342) and scrap metal (78/144), respectively. Among the other potential dengue vectors in the area, *Ae. furcifer* was collected from bricks (only one specimen), *Ae. luteocephalus* in tires (2 specimens), and *Ae. vittatus* mainly in bricks (12/15; 80%).

**Table 1:** Mosquito species composition in different resting places, southeastern Senegal, 2018

**Species**	Bricks		Rooms		Scrap metal		Tires		Total	
	*n*	%	*n*	%	*n*	%	*n*	%	*n*	%
*Ae. aegypti*	110	41.8	320	59.0	78	54.2	174	50.9	682	52.8
*Ae. argenteopunctatus*	0	0	0	0	1	0.7	0	0	1	0.1
*Ae. furcifer*	1	0.4	0	0	0	0	0	0	1	0.1
*Ae. luteocephalus*	0	0	0	0	0	0	2	0.6	2	0.2
*Ae. vittatus*	12	4.6	1	0.2	1	0.7	1	0.3	15	1.2
*An. coustani*	0	0	1	0.2	0	0	0	0	1	0.1
*An. domicola*	1	0.4	0	0	0	0	0	0	1	0.1
*An. funestus*	1	0.4	0	0	0	0	0	0	1	0.1
*An. gambiae*	4	1.5	11	2.0	7	4.9	5	1.5	27	2.1
*An. rufipes*	2	0.8	2	0.4	0	0	2	0.6	6	0.5
*Cx. cinereus*	2	0.8	0	0	0	0	4	1.2	6	0.5
*Cx. decens*	3	1.1	10	1.8	1	0.7	10	2.9	24	1.9
*Cx. neavei*	0	0	1	0.2	0	0	1	0.3	2	0.2
*Cx. nebulosus*	6	2.3	10	1.8	4	2.8	5	1.5	25	1.9
*Cx. perfuscus*	9	3.4	8	1.5	15	10.4	8	2.3	40	3.1
*Cx. quinquefasciatus*	108	41.1	176	32.5	35	24.3	127	37.1	446	34.5
*Cx. tigripes*	3	1.1	0	0	1	0.7	3	0.9	7	0.5
*Mansonia uniformis*	0	0	0	0	1	0.7	0	0	1	0.1
*Mimomiya splendens*	1	0.4	0	0	0	0	0	0	1	0.1
*Uranotaenia mayeri*	0	0	2	0.4	0	0	0	0	2	0.2
Total	263		542		144		342		1291	

*Abbreviations*: *n*, number collected; %, percent of the total

### Mosquito density in different localities and resting places

**Fig. 2:**
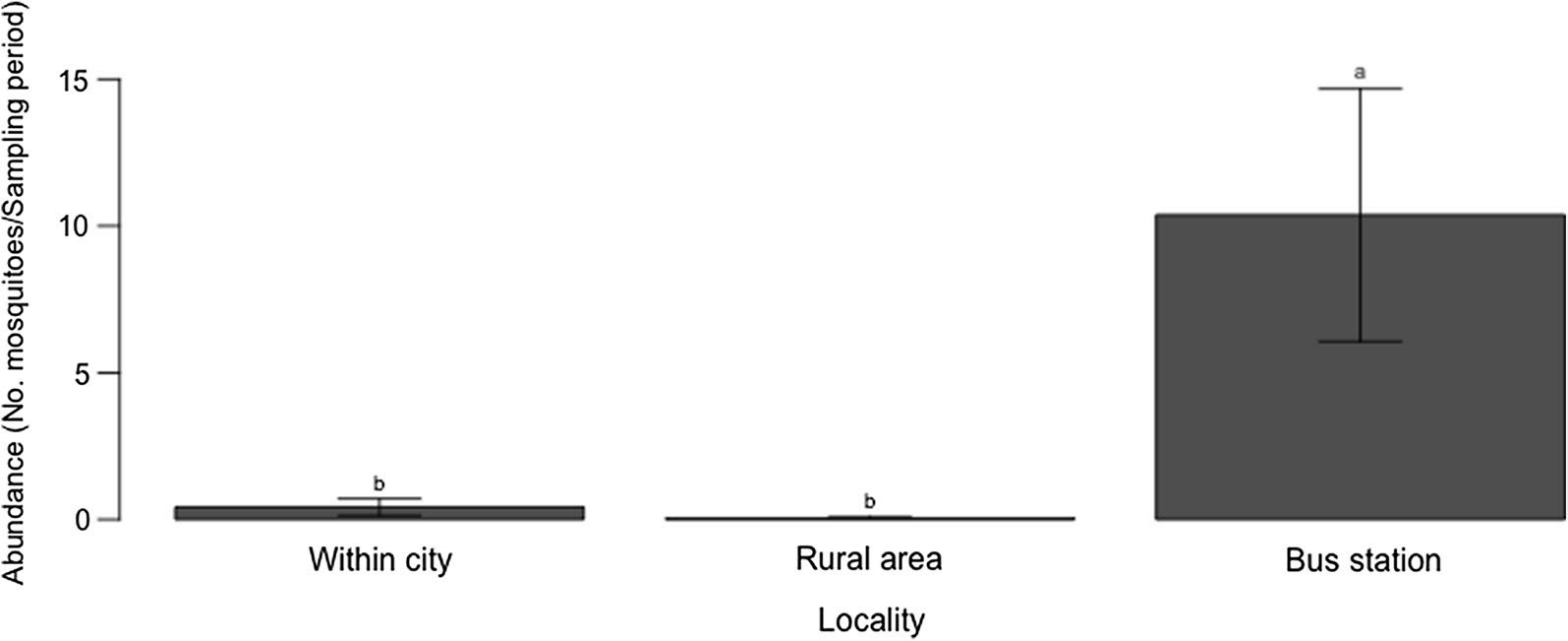
Average numbers of *Ae. aegypti* collected at the different localities in the Kedougou area, Senegal, 2018. Letters indicate the results of the pairwise Dunn’s test when the Kruskal-Wallis test was found statistically significant. Groups that do not share a letter are significantly different (*P* < 0.05)

The average number of *Ae. aegypti* collected at the different localities varied significantly (Kruskal-Wallis H-test: *χ*^2^ = 74.3, *df* = 2, *P* < 0.0001) (Fig. 2). This number was significantly higher at the bus station (10.4 ± 13.6) than in other areas within the city (0.4 ± 0.9) and in rural areas (0.03 ± 0.2) (*P* < 0.0001). The last two were comparable (*P* = 0.48).

**Fig. 3:**
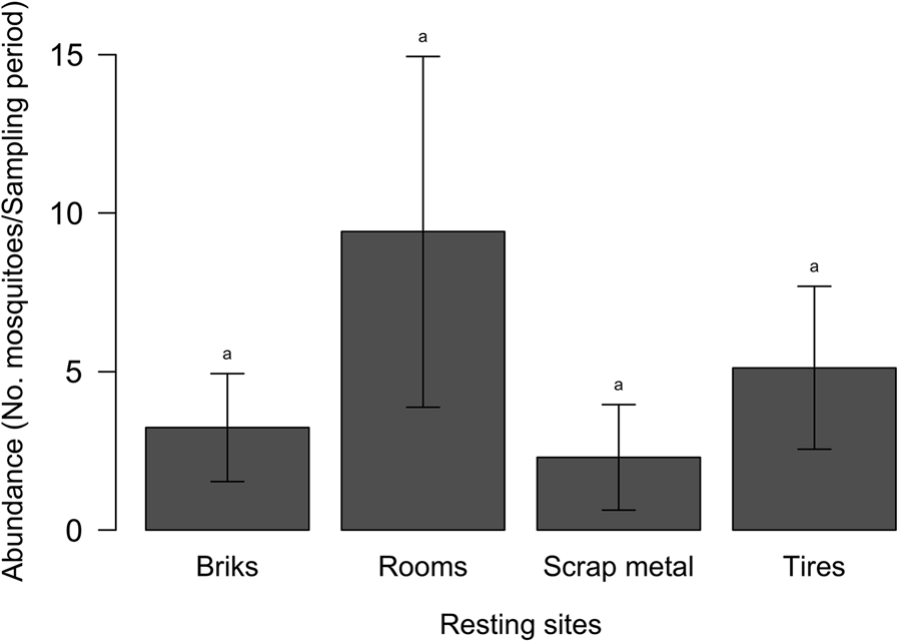
Average numbers of *Ae. aegypti* collected at different resting places in the Kedougou area, Senegal, 2018. Letters indicate the results of the pairwise Dunn’s test when the Kruskal-Wallis test was found statistically significant. Groups that do not share a letter are significantly different (*P* < 0.05)

Although the average numbers of mosquitoes collected in the different resting places varied between 9.4 ± 17.5 mosquitoes per site per collection time for rooms and 2.3 ± 5.3 for scrap metal (Fig. 3), all the resting places were equally used by *Ae. aegypti* (Kruskal-Wallis H-test: *χ*^2^ = 5.3, *df* = 3, *P* = 0.151).

### Sex and blood feeding status of *Aedes aegypti* females in different resting places

The percentage of resting *Ae. aegypti* females (Table 2), which varied between 38.1% in rooms and 59.2% in tires, presented statistically significant differences (Chi-square test: *χ*^2^ = 20.9, *df* = 3, *P* = 0.0001). Differences in percentages of females were due to the lower percentage of females in the population of *Ae. aegypti* resting in rooms; the others being comparable (Chi-square test: *χ*^2^ = 5.12, *df* = 2, *P* = 0.08).

**Table 2:** Number and relative abundance of *Aedes aegypti* by sex and abdominal status collected in different resting places, southeastern Senegal, 2018

Status of resting *Ae. aegypti*	Bricks		Rooms		Scrap metal		Tires		Total	
	*n*	%	*n*	%	*n*	%	*n*	%	*n*	%
Abdominal status of females										
Unfed	34	61.8^a^	40	32.8^b^	19	54.3^a^	68	66.0^a^	161	51.1
Blood-fed	20	36.4^b^	77	63.1^a^	14	40.0^b^	26	25.2^c^	137	43.5
Gravid	1	1.8^a^	5	4.1^a^	2	5.7^a^	8	7.8^a^	16	5.1
Semi-gravid	0	0	0	0	0	0	1	1.0	1	0.3
Sex										
Females	55	50.0^a^	122	38.1^b^	35	44.9^a^	103	59.2^a^	315	46.2
Males	55	50.0	198	61.9	43	55.1	71	40.8	367	53.8
Total	110		320		78		174		682	

*Notes*: letters in each group indicate the results of Chi-square test. Groups that do not share a letter are significantly different (*P* < 0.05)

*Abbreviations*: *n*, number collected; %, percent of the total

The rates of unfed females (Table 2) also varied significantly (Chi-square test: *χ*^2^ = 28.2, *df* = 3, *P* < 0.0001). Differences were due to the lower rate observed in rooms. The others were statistically comparable (Chi-square test: *χ*^2^ = 1.56, *df* = 2, *P* = 0.45). The rates of fed females in rooms (63.1%) and tires (25.2%) were respectively significantly higher and lower than those observed in bricks and scrap metal (Chi-square test: *χ*^2^ = 15.3, *df* = 3, *P* = 0.002). The latter were comparable (Chi-square test: *χ*^2^ = 0.01, *df* = 1, *P* = 0.9). The proportions of gravid females collected in the different resting places, which varied between 7.8% in tires and 1.8% in rooms were comparable (Fischer exact test: *P* = 0.7).

## Discussion

Knowledge of the resting behavior of *Ae. aegypti*, the main vector of dengue, chikungunya, yellow fever and Zika worldwide, is of considerable epidemiological importance. The firm identification of exact resting places of *Ae. aegypti* could allow the focal spraying of insecticides in these sites in an environmental and economically supportable ways, thus managing the insecticides resistance in mosquitoes and reducing theirs impact on non-target organisms. This reasonable way of using insecticides will help the low-income countries, that are among the most affected by arboviral diseases, to mitigate outbreaks despite theirs lack of human, logistical and financial resources. The identification of *Ae. aegypti* resting places makes also easier the surveillance of this vector and the associated arboviruses. Indeed, it allows the rapid collection of huge populations of this species for virus isolation attempts (for males, unfed and gravid females) or host feeding patterns, and identification of potential vertebrate reservoirs in arbovirus studies (for blood-fed females). The identification of these resting places could also be used for the study of the dispersal of the species by easily collecting the marked specimens in these sites placed in known locations and distance from the release site.

*Aedes aegypti* is traditionally known as a very endophilic species that rests almost exclusively inside human habitations [9, 10, 23, 24]. However, some authors also considered the possibility for this species to rest outdoors in dark and shady areas near its breeding sites [12]. The results of this study showed clearly a population of *Ae. aegypti* resting outdoors inside these breeding sites, such as used tires and bricks. Thus, used tires and bricks should be considered as both breeding sites and resting places of *Ae. aegypti* in southeastern Senegal. Further investigations are needed to see if the populations of this species from other localities in Senegal and elsewhere have the same resting behavior.

The use of outdoor resting places (tires, bricks and scrap metals) by *Ae. aegypti* has important implications for vector control strategies, particularly in an outbreak response context. Thus, it seems essential to target these outdoor resting places during insecticides aerial spraying. The application of insecticides to prevent *Aedes* breeding in tires as previously used [25], could be effective to control both immature and adult populations of *Ae. aegypti.* Further studies are needed to see if this strategy could prevent or control arbovirus outbreaks in the urban context.

The higher average density of *Ae. aegypti* at the bus station compared to other parts of the city and the rural environment is related to a higher number of used tires present in this environment. Used tires are known as good breeding sites for this species [26, 27], and in this study we showed that they were also very good resting places for *Ae. aegypti*. The occurrence of this originally sylvatic *Ae. aegypti* population [16] in used tires suggest a progressive adaptation of this mosquito strain in the Kedougou urban area [14]. Because humans are the main vertebrate species present in this area, this population of *Ae. aegypti* was very aggressive to people. Moreover, other important sylvatic vectors of arboviruses in the area, such as *Ae. furcifer* and *Ae. luteocephalus*, were found in low numbers in these resting places, suggesting that they could also adapt to the urban domestic environment. Therefore, they could play a key role in transmission and change the epidemiology of these arboviruses in southeastern Senegal. The detection of these sylvatic species in bricks and tires also suggests that they could potentially be used as resting traps for these species in the forests.

Currently human landing collection, targeting the host-seeking females, is the main method used for the entomological surveillance of *Ae. aegypti* and the *Aedes* borne viruses. Because this method presents many drawbacks (it is labor intensive, time consuming and ethically questionable), several alternative methods, including the collection of resting adults were proposed. The main problem of collecting resting adults was the firm identification of the resting places which are usually unknown or not accessible [13]. The great diversity of mosquito species encountered in these study areas and the high density of the population of *Ae. aegypti* suggest that collecting resting mosquitoes in used tires and bricks could be a good and cheap entomological surveillance method. Indeed, collecting resting mosquitoes in these newly discovered resting places (used tires and bricks) by aspiration could enable collection, easily and rapidly, of a huge number of mosquitoes from a different physiological status.

## Conclusions

This study showed that *Ae. aegypti* could be found resting indoors and in several sites, including used outdoor tires. This is, to our knowledge, the first description of a population of this species resting mainly outdoors not only around but mainly inside its breeding sites such as used tires and bricks. These data will be helpful in setting better arbovirus surveillance and vector control strategies.

## Data Availability

All data generated or analysed during this study are included in this published article.
